# Computed tomography-based psoas skeletal muscle area and radiodensity are poor sentinels for whole L3 skeletal muscle values^☆^

**DOI:** 10.1016/j.clnu.2019.10.003

**Published:** 2020-07

**Authors:** Katie E. Rollins, Aravin Gopinath, Amir Awwad, Ian A. Macdonald, Dileep N. Lobo

**Affiliations:** a Gastrointestinal Surgery, Nottingham Digestive Diseases Centre and National Institute for Health Research (NIHR) Nottingham Biomedical Research Centre, Nottingham University Hospitals NHS Trust and University of Nottingham, Queen's Medical Centre, Nottingham, NG7 2UH, UK; b University of Nottingham Medical School, Queen's Medical Centre, Nottingham, NG7 2UH, UK; c Sir Peter Mansfield Imaging Centre (SPMIC), University of Nottingham, University Park, Nottingham, NG7 2RD, UK; d School of Life Sciences, University of Nottingham, Queen's Medical Centre, Nottingham NG7 2UH, UK; e MRC Versus Arthritis Centre for Musculoskeletal Ageing Research, School of Life Sciences, University of Nottingham, Queen's Medical Centre, Nottingham, NG7 2UH, UK

**Keywords:** Computed tomography, Psoas, Skeletal muscle, Sarcopenia, Hounsfield unit, Density

## Abstract

**Background and aims:**

Computed tomography (CT)-based measurement of skeletal muscle cross-sectional area (CSA) and Hounsfield unit (HU) radiodensity are used to assess the presence of sarcopenia and myosteatosis, respectively. The validated CT-based technique involves analysis of skeletal muscle at the third lumbar vertebral (L3) level. Recently there has been increasing interest in the use of psoas muscle alone as a sentinel. However, this technique has not been extensively investigated or compared with the previous validated standard approach.

**Methods:**

Portovenous phase CT images at the L3 level were identified retrospectively from a single institution in 150 patients who had non-emergency scans and were analysed by a single assessor using SliceOmatic software v5.0 (TomoVision, Canada). Manual segmentation based upon validated HU thresholds for skeletal muscle density was performed for all skeletal muscle, as well as the individual muscle groups. The muscle CSA and mean radiodensity of each group were compared against the whole L3 slice values.

**Results:**

When compared with whole L3 slice CSA, anterior abdominal wall CSA had the strongest correlation (r = 0.9315, p < 0.0001) followed by paravertebral (r = 0.8948, p < 0.0001), then psoas muscle (r = 0.7041, p < 0.0001). The mean ± SD density of the psoas muscle (42 ± 8.4 HU) was significantly higher than the whole slice radiodensity (32.3 ± 9.5 HU, p < 0.0001), with paravertebral radiodensity being a more accurate estimation (34.5 ± 10.8 HU). There was a significant difference in the prevalence of myosteatosis when the density measured from the psoas was compared with that of the whole L3 skeletal muscle (27.7% vs. 66.0%, p < 0.0001).

**Conclusion:**

Whole L3 slice CSA correlated positively with psoas muscle CSA but was subject to wide variability in results. Psoas muscle radiodensity was significantly greater than whole L3 slice density and resulted in underestimation of the prevalence of myosteatosis. Given the lack of equivalence from individual muscle groups, we recommend that further work be undertaken to investigate which muscle group, or indeed whether the gold standard of whole L3 skeletal muscle, provides the best correlation with clinical outcomes.

## 1 Introduction

Body composition analysis using computed tomography (CT) images to determine the presence of sarcopenia and myosteatosis has become a popular area of research. Studies have found a link between the presence of sarcopenia (more notably sarcopenic obesity) and myosteatosis (attenuated skeletal muscle density) and impaired tolerance to chemotherapy [1,2], increased incidence of postoperative complications [3,4], as well as reduced overall survival [5,6] in a range of cancer subtypes. The generally accepted definition for these body composition variables from CT imaging is based upon values calculated at the third lumbar vertebral level (L3) using validated Hounsfield Unit (HU) thresholds to manually segment tissue regions of interest. For sarcopenia, this is defined by the skeletal muscle index (SMI) which normalises the whole L3 skeletal muscle cross-sectional area (CSA) by the patients' height squared. The threshold for the diagnosis of sarcopenia is variable and is determined by both patient gender and BMI. The presence of myosteatosis is determined from the mean skeletal muscle HU density and is similarly dependent on BMI [7].

However, several methodological issues with assessment of body composition using CT have been highlighted recently. These include variability according to the phase of contrast of the scanned images [[8], [9], [10]] and the software utilised [[11], [12], [13]]. In addition, several studies, representing approximately 6% of the literature using CT image slices [14], have investigated the role of single muscle groups rather than whole cross-sectional slice variables. Reduced overall psoas muscle CSA [15,16] and lower muscle density [17,18] have been associated with a negative prognosis in gastrointestinal malignancy, as has paravertebral radiodensity in patients with gastrointestinal cancer with spinal metastases [19]. The rationale for the use of the psoas muscle for body composition analysis has been that this is ‘simple and convenient’ to measure [20]. However, there are large discrepancies in the use of psoas muscle area as a measure of sarcopenia including normalisation for patient height, body surface area and to the area of the adjacent vertebral body. In terms of validation of different muscle groups with whole L3 slice variables, a study undertaken in the field of ovarian cancer [21] compared skeletal muscle area between whole lumbar cross sectional values and psoas muscle. This demonstrated poor correlation between the two and concluded that total skeletal muscle area at L3 was a superior predictor of overall survival in this cohort. This study [21] also noted that the psoas muscles represented less than 10% of the whole trunk muscles and had high levels of discrepancy in measurement. In a cohort of 353 patients on the waiting list for liver transplant due to cirrhosis [22], the psoas muscle index was a far poorer predictor of mortality than whole L3 slice SMI. A recent study using psoas muscle density has also confused myosteatosis with sarcopenia [23]. These inconsistencies make it difficult to interpret the data across studies and lead to a degree of methodological confusion.

No study has previously examined the relationship of skeletal muscle HU density calculated for the whole slice versus different individual skeletal muscle groups at the L3 level, nor has there been further investigation regarding the relationship between whole L3 slice CSA and the CSA of different individual skeletal muscle groups.

The aims of this study were, therefore, to:

- examine the relationship between whole slice, psoas muscle, paravertebral and anterior abdominal wall CSA
- examine the relationship between whole slice, psoas muscle, paravertebral and anterior abdominal wall muscle mean HU density and the impact of this upon the prevalence of myosteatosis.

## 2 Methods

A series of 150 patients who had undergone portovenous phase abdominal CT scans including the L3 level at Nottingham University Hospitals NHS Trust were included in the study. All scans were performed for routine clinical purposes and were identified retrospectively from the hospital radiology records. Additional data on patient demographics including height and weight from within one month of the scan, age and gender were obtained from the hospital electronic records. The study was registered with the Audit Department of Nottingham University Hospitals.

### 2.1 Body composition analysis

Axial CT scan images at the L3 vertebral level were obtained from the Picture Archiving and Communication System (PACS) in Digital Imaging and Communication in Medicine (DICOM) format for all patients. A single trained investigator analysed all the CT images using SliceOmatic software version 5.0 (TomoVision, Montreal, Canada). The previously validated Hounsfield unit (HU) densities of skeletal muscle of −29 to +150 HU [24], visceral adipose tissue of −150 to −50 [25] and subcutaneous and intramuscular adipose tissue of −190 to −30 [26] were used to manually segment the skeletal muscle within the different muscle groups. The muscle groups individually analysed were: whole L3 slice, psoas, paravertebral and anterior abdominal wall, with each group divided into left and right-sided muscle groups which were also compared. The software automatically calculates the cross-sectional skeletal muscle area in cm^2^ as well as the mean HU density. The patients' height was used to calculate the skeletal muscle index (SMI) from the whole slice cross-sectional skeletal muscle area. The threshold radiological values for the diagnosis of myosteatosis were taken from the validated values for the whole L3 slice as there are currently no validated thresholds for individual muscle groups. These were defined operationally as a mean skeletal muscle radiodensity of <41 HU in those with BMI <25 kg/m^2^ and <33 HU in those with a BMI ≥25 kg/m^2^ [7].

### 2.2 Statistical analysis

Data were analysed using GraphPad Prism v8.0 (GraphPad, La Jolla, California, USA). Data were assessed for normality using the D'Agostino-Pearson normality test. Parametrically distributed data were presented as mean ± standard deviation and non-parametrically distributed data presented as median (interquartile range). Mean skeletal muscle HU densities between different muscle groups were compared with paired t-testing and correlation analysis was performed using Pearson's correlation coefficient. This correlation testing was also applied to comparisons of skeletal muscle area from different muscle groups. Linear regression equations were planned in order to extrapolate whole L3 slice CSA and mean HU radiodensity. Finally, the prevalence of myosteatosis was compared according to which skeletal muscle radiodensity was used for the analysis and the relative incidences compared. All analyses were performed using two-tailed testing and significance was indicated by a p value < 0.05.

## 3 Results

A total of 150 patients who had undergone CT scans between September 2011 and December 2017 were included in the study. There were 58 females and 92 males, with a mean age of 65.8 ± 9.5 years and a mean BMI of 27.31 ± 5.54 kg/m^2^. Mean skeletal muscle index (SMI) taken from the whole L3 slice was 48.31 ± 10.30 and mean skeletal muscle HU density was 32.26 ± 9.50 HU. The mean visceral and subcutaneous/intramuscular adipose cross-sectional area for the cohort was 148.72 ± 97.55 cm^2^ and 229.76 ± 124.30 cm^2^ respectively.

### 3.1 Skeletal muscle CSA

**Fig. 1:**
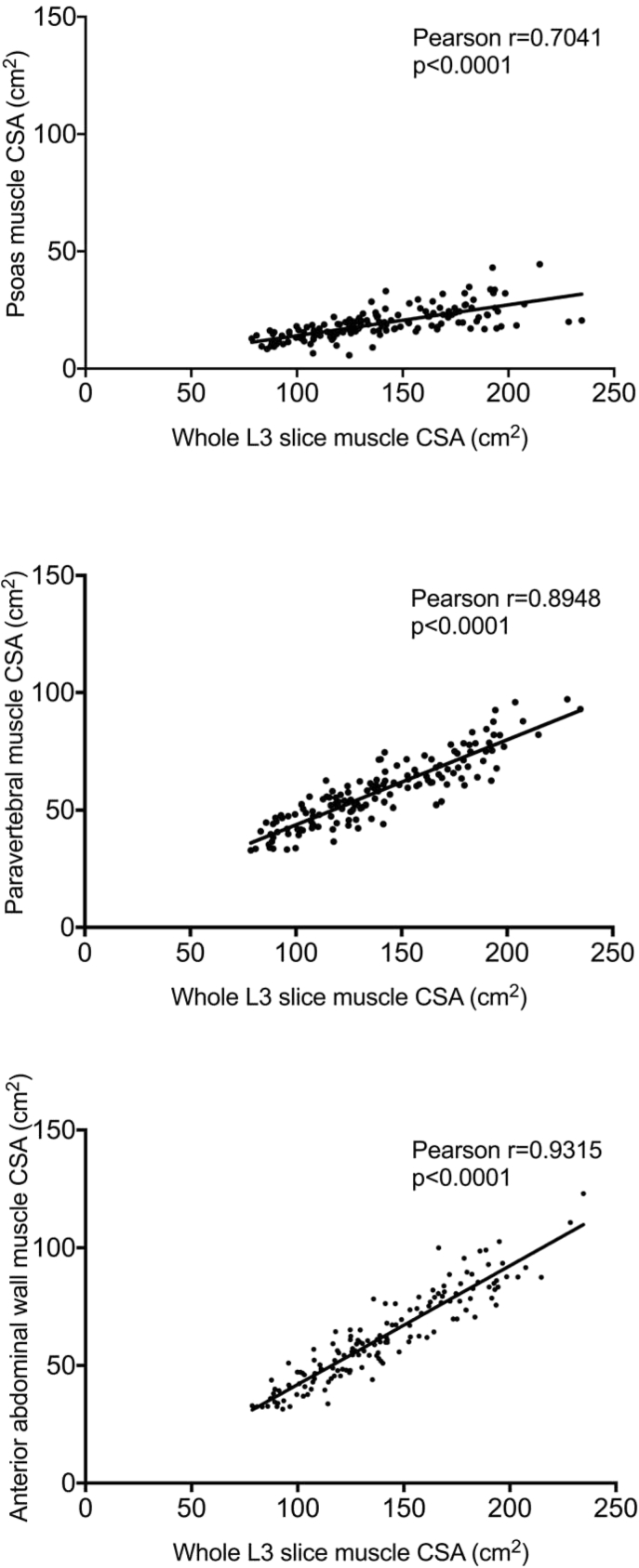
Correlation between whole L3 slice muscle cross sectional area (CSA) and individual skeletal muscle group CSA. top – whole slice vs. psoas, middle – whole slice vs. paravertebral, bottom – whole slice vs. anterior abdominal wall.

Overall, the mean whole L3 slice CSA was 139.28 ± 35.25 cm^2^, the psoas CSA was 19.22 ± 6.58 cm^2^, paravertebral muscle CSA was 57.91 ± 14.22 cm^2^ and the anterior abdominal wall muscle CSA was 61.70 ± 19.09 cm^2^. There was a strong positive correlation between whole L3 slice muscle CSA and individual muscle group CSA (Fig. 1), although the most positive association was between whole slice and anterior abdominal wall muscle groups (Pearson r = 0.9315, p < 0.0001) and least was the psoas muscle CSA (Pearson r = 0.7041, p < 0.0001), with a much greater spread of results. Given the degree of variability and systematic bias in these results, the decision was made not to generate linear regression equations for the calculation of whole L3 slice CSA from individual muscle groups.

### 3.2 Right versus left sided muscle group CSA

When individual muscle groups were divided into right and left sides, there was no significant difference in CSA in the psoas and whole slice analysis (Table 1), indicating equivalence between the two sides. However, there was a statistically significant difference in the CSA of the right and left sided paravertebral and anterior abdominal wall muscle groups, although the clinical significance of this difference is unclear, representing just 2.2% and 1.3% of the total muscle areas respectively.

**Table 1:** Comparison between right and left muscle groups for individual skeletal muscle cross sectional areas (CSA) (cm^2^).

Skeletal Muscle Variable	Psoas CSA (cm^2^)	Paravertebral CSA (cm^2^)	Anterior Abdominal Wall CSA (cm^2^)	Whole Slice SM CSA (cm^2^)
Right side mean ± SD	9.7 ± 3.4	28.5 ± 7.2	31.3 ± 9.8	69.3 ± 17.6
Left side mean ± SD	9.7 ± 3.7	29.8 ± 7.5	30.5 ± 9.7	69.9 ± 18.0
Mean difference	−0.002 p = 0.099	1.3 p < 0.0001	−0.8 p = 0.013	0.6 p = 0.20

### 3.3 Mean skeletal muscle HU radiodensity

**Fig. 2:**
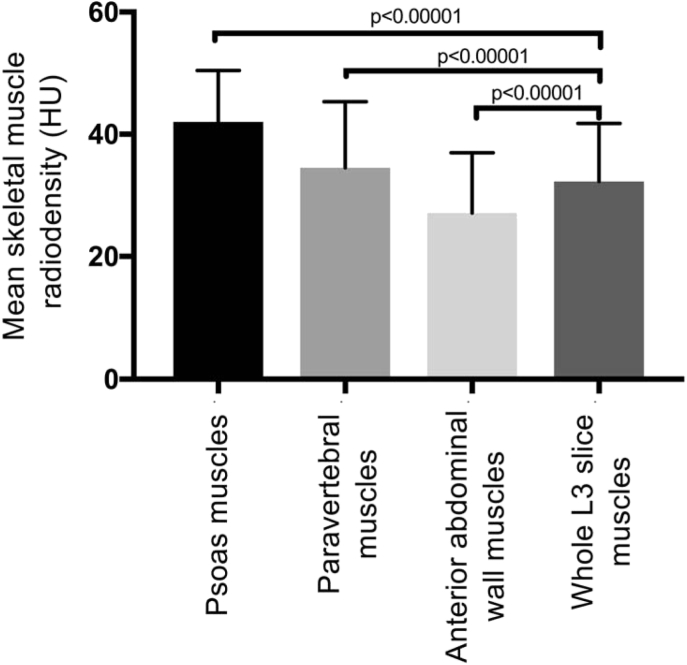
Comparison between whole L3 slice radiodensity and individual skeletal muscle groups.

**Fig. 3:**
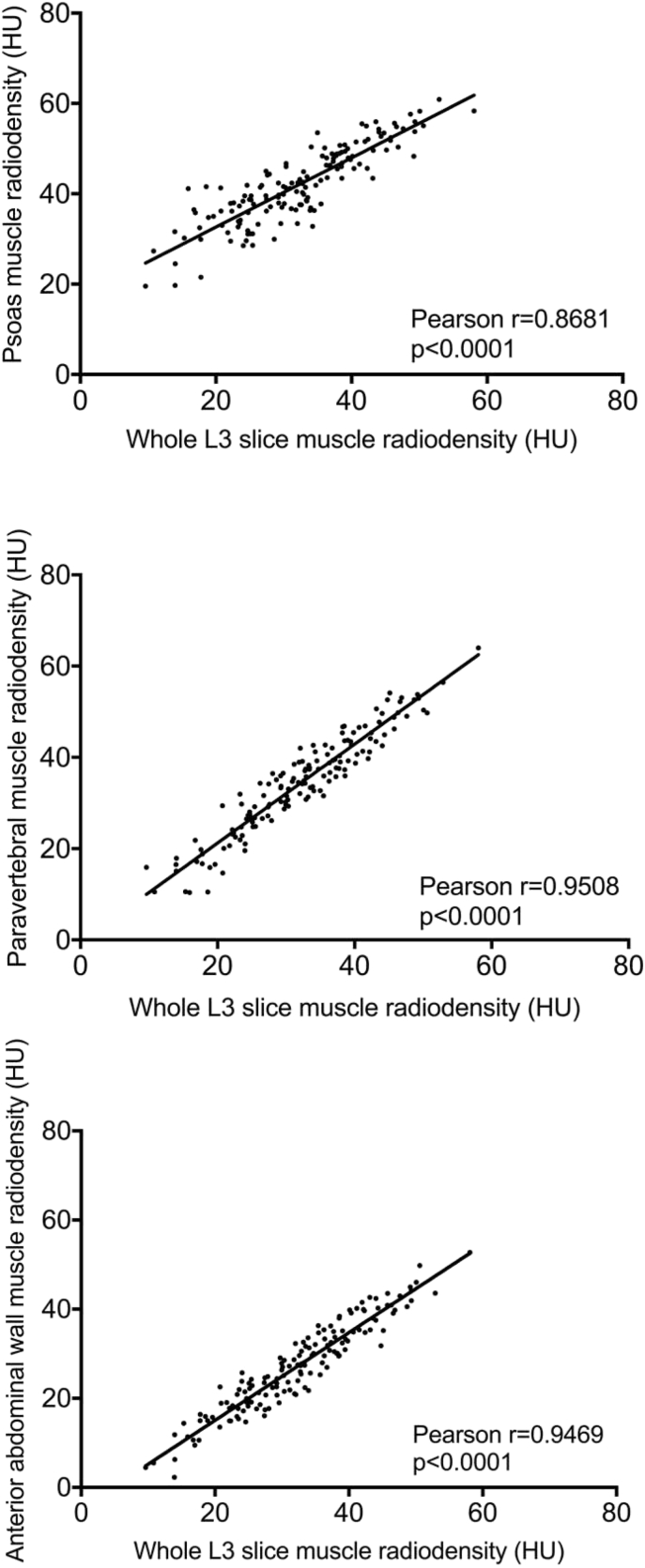
Correlation between whole L3 slice radiodensity and individual skeletal muscle radiodensity. top – whole slice vs. psoas, middle – whole slice vs. paravertebral, bottom – whole slice vs. anterior abdominal wall.

There were significant differences in the mean skeletal muscle radiodensity between individual skeletal muscle groups and the whole L3 slice, with the highest density observed in the psoas muscle (42.0 ± 8.4 HU), followed by the paravertebral muscle (34.5 ± 10.8 HU), the whole L3 slice (32.3 ± 9.5 HU) and finally the anterior abdominal wall muscle with the lowest density (27.1 ± 9.9 HU) (Fig. 2). The paravertebral muscle group was the closest to the validated standard approach of whole L3 slice skeletal muscle density, with a mean difference of 2.2 HU. However, this remained statistically significantly greater than whole slice muscle radiodensity. There was a strong positive correlation between the whole L3 slice radiodensity and that of the other individual muscle groups, all of which were statistically significant (Fig. 3), with the closest relationship being between whole L3 slice and paravertebral density (Pearson r = 0.9508, p < 0.0001).

### 3.4 Prevalence of myosteatosis

The prevalence of myosteatosis when the radiodensity was taken from the whole L3 slice was 66.0%. However, when this was calculated according to psoas muscle density, the prevalence was 27.7%, representing both a clinically and statistically significant difference (p < 0.0001). Similarly, the prervalence of myosteatosis was lower when the density of the paravertebral muscles was used (56.6%). However, this difference was not statistically significant (p = 0.138). The prevalence of myosteatosis estimated by the density of the anterior abdominal wall muscles was significantly higher than that of the whole L3 slice (79.0% vs. 66.0%, p = 0.017).

## 4 Discussion

This study has demonstrated that psoas muscle CSA correlated positively with whole L3 slice skeletal muscle CSA, but the correlation was not as high when paravertebral or anterior abdominal wall muscles were correlated with the whole L3 slice. With regards to skeletal muscle density, psoas muscle had a significantly higher radiodensity than whole L3 slice skeletal muscle, with paravertebral muscle representing the closest approximation of the validated standard approach measure of whole L3 slice radiodensity, and the radiodensity of the anterior abdominal wall muscles was significantly lower than that of the whole L3 slice. This resulted in vastly differing results in terms of the prevalence of myosteatosis within the cohort when the validated thresholds were utilised. The closest muscle group in terms of skeletal muscle radiodensity of the whole L3 slice, representing the validated standard approach, was the paravertebral muscles.

The results of this study are similar to those of a previous study which examined the relationship between psoas and whole L3 slice CSA in ovarian malignancy [21]. The previous study [21] found weakly positive inter-measurement correlation between psoas and whole L3 slice CSA of 0.52 prior to chemotherapy and 0.56 following chemotherapy, less strong than the correlation observed in the current study. These authors did not consider skeletal muscle radiodensity between the different muscle groups. However, the study [21] explored several potential reasons for the large discrepancy in skeletal and psoas muscle CSA, with a particular emphasis on the preferential wasting of the psoas muscle in degenerative diseases of the lumbar spine, frequently seen in the elderly. Note should be made of the different physiological functions of the individual skeletal muscle groups within this study. The psoas muscle is the main flexor of the hip joint and key to maintaining posture and mobility, hence during periods of immobilisation the psoas muscle is known to decrease in size which may represent global muscle loss in patients. The anterior abdominal wall muscles act as a durable and flexible cover for the abdominal viscera, assist in respiration, coughing and vomiting by changes in intra-abdominal pressure and facilitate flexing of the trunk. Finally, the paravertebral muscles are involved in extension of the spine, maintenance of posture and global spinal alignment. Therefore, there may be a degree of discrepancy in CSA of different muscle groups which is related to the patients' underlying mobility and their comorbid status. There is additional evidence from animal studies that these individual muscle groups differ in their sensitivity to insulin [[27], [28], [29]]. Research in humans suggests a positive correlation between proportions of slow, oxidative type I fibres in muscle and whole-body insulin sensitivity [[30], [31], [32]]. Hence, type I muscle fibres are more insulin sensitive than type II fibres. Insulin-stimulated glucose transport in human muscle was proportional to the relative type I fibre content [33]. Human type I fibres may, therefore, be more important than type II fibres for maintaining glucose homeostasis in response to insulin. A decreased proportion of type I fibres has been found in various insulin resistant states such as the metabolic syndrome, obesity and in some patients with type 2 diabetes [34]. This phenomenon is also seen following bed rest, as well as in tetraplegic patients, and those with an insulin receptor gene mutation [34]. This may, to some extent, explain the differences in radiodensities in the various muscle groups studied.

Clearly differences are expected in the overall CSA between individual muscle groups and the whole L3 slice, however the correlation between the whole slice and psoas muscle was far from straightforward, with a degree of variability which became more pronounced in those patients with an increased whole L3 skeletal muscle CSA. Given the lack of validation of a SMI particular to the psoas muscle, no analysis was performed to compare whole L3 slice SMI with that of the psoas muscle. The difference in skeletal muscle density was, however, very striking and the demonstration that psoas and whole slice radiodensity are not at all comparable is clinically relevant. Whole L3 slice HU radiodensity must remain the standard approach for the assessment of the presence of myosteatosis. Given the process for calculation of mean skeletal muscle density involves manual segmentation of all skeletal muscle from the L3 slice, no further work is required for the software to additionally calculate the CSA from this manually segmented area, thus it would seem obvious that both measures should be calculated from the same region of interest.

This study was performed retrospectively using a non-selected patient cohort. As such, there are limitations to some of the data obtained. This includes height and weight data only being known for within one month of the date of the CT scan, no knowledge surrounding the hydration status of the patient at the time of their CT scan and no data regarding the stability of patients' nutritional state. Given the link between spinal pathology and preferential wasting of the psoas muscle, it is notable that no history was available on whether the patients had a history of spinal pathology or chronic lower back pain.

If there remains an ongoing push for single muscle group analysis, despite increasing evidence that this is a suboptimal technique for body composition analysis, robust evidence from prospective studies would be necessary comparing the association between negative treatment outcomes in patients with cancer with both whole L3 slice, psoas and paravertebral muscle CSA and radiodensity. This would provide confirmation which is the best prognosticator for worse clinical outcomes.

In addition, it is important to emphasise the pathophysiology behind the variations in muscle radiodensities. Muscle radiodensities on CT scanning are dependent on cardiac output at the time of scan and varied proportions of intramuscular vascular blood supply to the individual muscles in accordance with pathophysiological (sarcopenia/myosteatosis) and health (physiological muscle function and disuse atrophy) status. Any degree of fatty infiltration of muscles will reduce their radiodensity as well (as fat being measured in negative values on HU radiodensity scales). Fatty infiltration can be assessed visually and quantified with fat suppression MRI techniques [35]. Different sequences are now available to choose from, and this will enable research to calculate pre and post suppression of intra-muscular (molecular) fat signal values within the muscles [36]. Thus, in the future, MRI may be a better modality to assess myosteatosis rather than CT [36,37].

## 5 Conclusion

The psoas CSA as measured by CT-based body composition analysis correlated with the whole L3 slice CSA. However, as this relationship is far from conclusive and is subject to a significant degree of variation, it should not be used as a sentinel for whole slice CSA. In addition, psoas muscle radiodensity cannot be used as a sentinel for whole L3 slice radiodensity, which is currently the validated standard approach for the diagnosis of myosteatosis. The use of the psoas muscle group alone leads to an underestimation of the prevalence of myosteatosis. Whilst whole L3 slice skeletal muscle area and radiodensity remain the gold standard for CT-guided body composition analysis, we recommend that individual muscle groups should not be used as a sentinel. However, given the demonstration that these individual muscle groups are highly disparate, particularly in terms of radiodensity, further research is recommended to correlate body composition analysis for the whole L3 slice as well as each individual skeletal muscle group with clinical outcome measures. This should be conducted with the aim of assessing which muscle group best correlates with relevant clinical outcome measures.

## Conflicts of interest

None of the authors has a direct conflict of interest to declare. IAM has received research funding from Mars Inc. and serves on the advisory board of IKEA, Nestle, Mars, Novozymes for unrelated work. DNL has received unrestricted research funding from B. Braun and speaker's honoraria from Fresenius Kabi, B. Braun, Shire and Baxter Healthcare for unrelated work.

## Funding

This work was supported by the 10.13039/501100000265Medical Research Council [grant number MR/K00414X/1]; and 10.13039/501100000341Arthritis Research UK [grant number 19891]. KER was supported by a Research Fellowship from the 10.13039/501100007743European Society for Clinical Nutrition and Metabolism (ESPEN).

## Author contributions

All authors contributed to the conception and design of the study collection, analysis or interpretation of data drafting the article or revising it critically for important intellectual content and final approval of the version to be published.
